# Differences on Prosaccade Task in Skilled and Less Skilled Female Adolescent Soccer Players

**DOI:** 10.3389/fpsyg.2021.711420

**Published:** 2021-10-14

**Authors:** Junyi Zhou

**Affiliations:** ^1^School of Physical Education and Sport Sciences, Fujian Normal University, Fuzhou, China; ^2^Provincial University Key Laboratory of Sport and Health Science, Fujian Normal University, Fuzhou, China; ^3^Key Laboratory of Kinesiological Evaluation General Administration of Sport of China, Fujian Normal University, Fuzhou, China

**Keywords:** saccadic eye movement, prosaccade task, cognitive processes, adolescent soccer players, skill level

## Abstract

Although the relationship between cognitive processes and saccadic eye movements has been outlined, the relationship between specific cognitive processes underlying saccadic eye movements and skill level of soccer players remains unclear. Present study used the prosaccade task as a tool to investigate the difference in saccadic eye movements in skilled and less skilled Chinese female adolescent soccer players. Fifty-six healthy female adolescent soccer players (range: 14–18years, mean age: 16.5years) from Fujian Youth Football Training Base (Fujian Province, China) took part in the experiment. In the prosaccade task, participants were instructed to fixate at the cross at the center of the screen as long as the target appeared peripherally. They were told to saccade to the target as quickly and accurately as possible once it appeared. The results indicated that skilled soccer players exhibited shorter saccade latency (*p*=0.031), decreased variability of saccade latency (*p*=0.013), and higher spatial accuracy of saccade (*p*=0.032) than their less skilled counterparts. The shorter saccade latency and decreased variability of saccade latency may imply that the attentional system of skilled soccer player is superior which leads to smaller attention fluctuation and less attentional lapse. Additionally, higher spatial accuracy of saccade may imply potential structural differences in brain underlying saccadic eye movement between skilled and less skilled soccer players. More importantly, the results of the present study demonstrated that soccer players’ cognitive capacities vary as a function of their skill levels. The limitations of the present study and future directions of research were discussed.

## Introduction

Eye-tracking methods are widely used in various research domains such as psychology, psychiatry, or sport (Duchowski, 2003). They are popular because eye movement measures are easy to obtain and participants can understand eye movement tasks easily (Noiret et al., 2017). More importantly, eye-tracking methods provide unique means to measure cognitive processes (Thomas et al., 2021). Therefore, eye movement experiments are carried out in different populations in various research domains (Noiret et al., 2018).

Saccade is a rapid eye movement we typically make three times every second (Rayner, 1998). Saccadic eye movements can be characterized by several eye movement measures such as saccade latency (i.e., the time elapsed from the onset of the target to the onset of the first saccade after target onset), saccade velocity (i.e., the velocity of saccade), and saccade amplitude (i.e., the spatial distance between two successive fixations). The prosaccade task is a well-known experimental paradigm that is used to examine saccadic eye movements. In a typical prosaccade task, participants are instructed to fix on a central dot. Then, they have to direct their gaze toward a target dot appearing at the periphery as quickly and as accurately as possible. Research has shown that saccadic eye movements are associated with multiple cognitive processes including processing speed, attention, and inhibitory control (Hutton, 2008; Noiret et al., 2017).

For instance, some previous studies have revealed that the saccade latency needed to trigger a prosaccade in prosaccade tasks varies as a function of the attentional process (Clark, 1999; Pratt et al., 2006; Noiret et al., 2018). Noiret et al. (2017) found that saccade latency is influenced by information processing speed. Moreover, some research on some special populations has suggested that saccade latency variability is a good indicator of attentional fluctuation in saccadic eye movement tasks (Kaufman et al., 2010, 2012). Kaufman et al. (2010) found that patients with Lewy body had greater saccade latency variability than aged controls. Mostofsky et al. (2001) examined the saccadic eye movements of boys with Tourette syndrome. They found that boys with ADHD (i.e., attention deficit hyperactivity disorder) had greater saccade latency variability than boys without ADHD in the prosaccade task. Studies on Alzheimer’s disease have shown that patients with Alzheimer’s disease had longer saccade latency as well as greater saccade latency variability than healthy senior adults.

The purpose of the present study was to use the prosaccade task as a tool to investigate the difference in cognitive processing underlying saccadic eye movements in skilled and less skilled female adolescent soccer players. Soccer is a game that requires players to process a great amount of internal and external information under pressure within a limited time and react to complex and rapidly changing contexts (Vestberg et al., 2012). Soccer players have to distribute their attention over multiple objects (e.g., moving ball, teammates, and opponents) during games (Ando et al., 2001). Therefore, saccadic eye movement is a critical index for soccer players’ performance. Some research has found that eye movement measures vary as a function of skill level in many sports. Ando et al. (2001) revealed that soccer players had shorter visual reaction time than nonathletes when responding to peripheral stimulus, indicating that soccer players had faster visual processing speed. Similarly, Zwierko (2008) found that handball players had shorter response time to peripheral stimulus compared to nonathletes. A recent study showed that cricketers had faster visual reaction times than nonathletes and cricketers’ stability of gaze is associated with faster visual reaction time (Barrett et al., 2020). Moreover, some researchers examined the search behaviors of skilled and less skilled soccer players when viewing film of defensive scenarios in soccer finding that skilled players exhibited more fixations, shorter duration, and more fixation locations than less skilled players (Ward and Williams, 2003; Williams and Ericsson, 2005; Williams, 2009; Roca et al., 2011).

In summary, previous studies have shown that there are differences in eye movement tasks among populations with various skill levels. However, to our knowledge, little work has used the prosaccade task to investigate the differences in saccadic eye movements between skilled and less skilled players. Some studies using the prosaccade task only focus on examining the cognitive changes of players caused by head impact exposure in contact and collision sports (Kiefer et al., 2018; Gallagher et al., 2020).

Therefore, the goal of the present study was to examine the potential differences in cognitive processes underlying saccadic eye movements between skilled and less skilled female adolescent soccer players by using the prosaccade task. Based on previous studies, we predict that skilled players would have higher cognitive ability and would thus perform better than less skilled players in prosaccade task. More specifically, we hypothesized that skilled players will exhibit shorter saccade latency, decreased variability of saccade latency, and higher spatial accuracy of saccade than their less skilled counterparts.

## Materials and Methods

### Ethics Statement

This study was approved by the Ethical Committee of the Fujian Normal University. All participants provided their written informed consent to participate in this study. This study was performed in full compliance with the Declaration of Helsinki.

### Participants

The G*Power tool (Erdfelder et al., 2009) was used to calculate the sample size in the present study. A statistical power analysis was conducted based on the reported effect size of skill level on saccade latency (mean Cohen’s *d*=0.817, from Ducrocq et al., 2016). The result indicated with an alpha level of 0.05; at least 46 participants are required to get a power of 0.80 (*n*=23 in each group). Therefore, we recruited 56 healthy female soccer players from Fujian Youth Football Training Base to participate in our experiment, which met the requirements of statistical power for replicating previous results. Their ages ranged from 14 to 18years, with an average of 16.5years. Each player was paid ¥50 for their participation. The experimental design of the present study is a one-way design. Players were assigned into two groups. The skilled player group consisted of 24 skilled players who were determined as Level 1 or Level 2 players. The less skilled player group consisted of 32 less skilled players who were determined as Level 3 or Level 4 players. The levels of players are determined by Fujian Provincial Sports Association according to the latest version of Technical Classification Standards of Soccer Players (2019 edition) developed by Chinese Football Association. Table 1 reports the age, training years, and the skill level ranking for skilled and less skilled players. Before participants were enrolled in the study, each of them was informed about the study protocol and gave his written informed consent. All participants had normal or corrected-to-normal vision, with no reported color blindness. None of them have suffered from any neurological or psychiatric disorders.

**Table 1:** Characteristics of skilled and less skilled adolescent soccer players.

	Skilled players (*n*=24)	Less skilled players (*n*=32)	*p*
Age (years)	16.6 (0.7)	16.4 (0.7)	0.197
Skill level ranking	1.5 (0.5)	3.5 (0.5)	<0.001
Training years	5.29 (0.62)	5.28 (0.58)	0.949

### Apparatus

The prosaccade task was programmed in Experimental Builder (SR Research Ltd.). The materials were presented on a 17-inch DELL PC laptop (DELL VOSTRO 15; resolution: 1,920×1,080 pixels; refresh rate: 150Hz). Stimulus was displayed in black (RGB: 0, 0, 0) on a grey background (RGB: 153, 153, 153). Participants were seated at a viewing distance of approximately 58cm from the computer monitor. A chin rest was used to stabilize the participants’ heads. Participants viewed stimulus binocularly, while only their right eyes were monitored. Their eye movements were recorded using an Eyelink Portable Duo eye-tracking system with a sampling rate of 1,000Hz.

### Procedure

The prosaccade task comprised 75 trials. Five of them were practice trials. Each trial began with a fixation cross (1°×1°) at the center of the screen displayed for 1,000ms. Then, the target circle (1.2°×1.2°) was displayed with an eccentricity of ±10° of visual angle in the horizontal plane for 1,500ms (30 trials for each side), followed by an intertrial interval randomly varied between 800 and 1,200ms. Participants were instructed to fixate at the cross to ensure that they were looking at the center of the screen when the target appeared peripherally. They were also instructed to look at the target circle as quickly and accurately as possible once it appeared.

Participants were tested individually in a quiet room. After reading the experimental instructions and a brief description of the apparatus, the chair was adjusted to make them feel comfortable, and the eye tracker was calibrated using a nine-point calibration and validation procedure. The maximal error of validation was below 0.5° in the visual angle. At the beginning of each trial, a black circle (0.5°×0.5°) was presented on the center of the computer screen as drift correction. Once the participant successfully fixated on the black circle, following stimuli were displayed. The prosaccade task lasted about 12min.

### Statistical Analysis

The raw eye movement data were analyzed using Data Viewer (SR Research Ltd.). To ensure the including eye movements data are qualified, following criteria for inclusion were used in analyses (Jazbec et al., 2006): (1) saccades with a latency between 80 and 800ms is observed, (2) saccade durations must be larger than 25ms, and (3) saccade amplitude must be greater than 3°. This resulted in a loss of approximately 11% of the trials.

On the basis of these criteria, the following saccade indexes were derived: (1) saccade latency, which was defined as the time elapsed from the onset of the target to the onset of the first saccade after target onset. (2) Variability of saccade latency, which was defined as the standard deviation of saccade latency. (3) Saccade amplitude, which was defined as the spatial distance between the last fixation on fixation cross and the first fixation on target circle. (4) Spatial accuracy of saccade, which was defined as the distance (in degrees of visual angle) between the landing position of the first saccade toward the target circle and the center of the target circle. (5) Peak velocity, which was defined as peak value of gaze velocity (in visual degrees per second) of the saccade. (6) Average velocity, which was defined as average velocity of the saccade.

## Results

**Figure 1:**
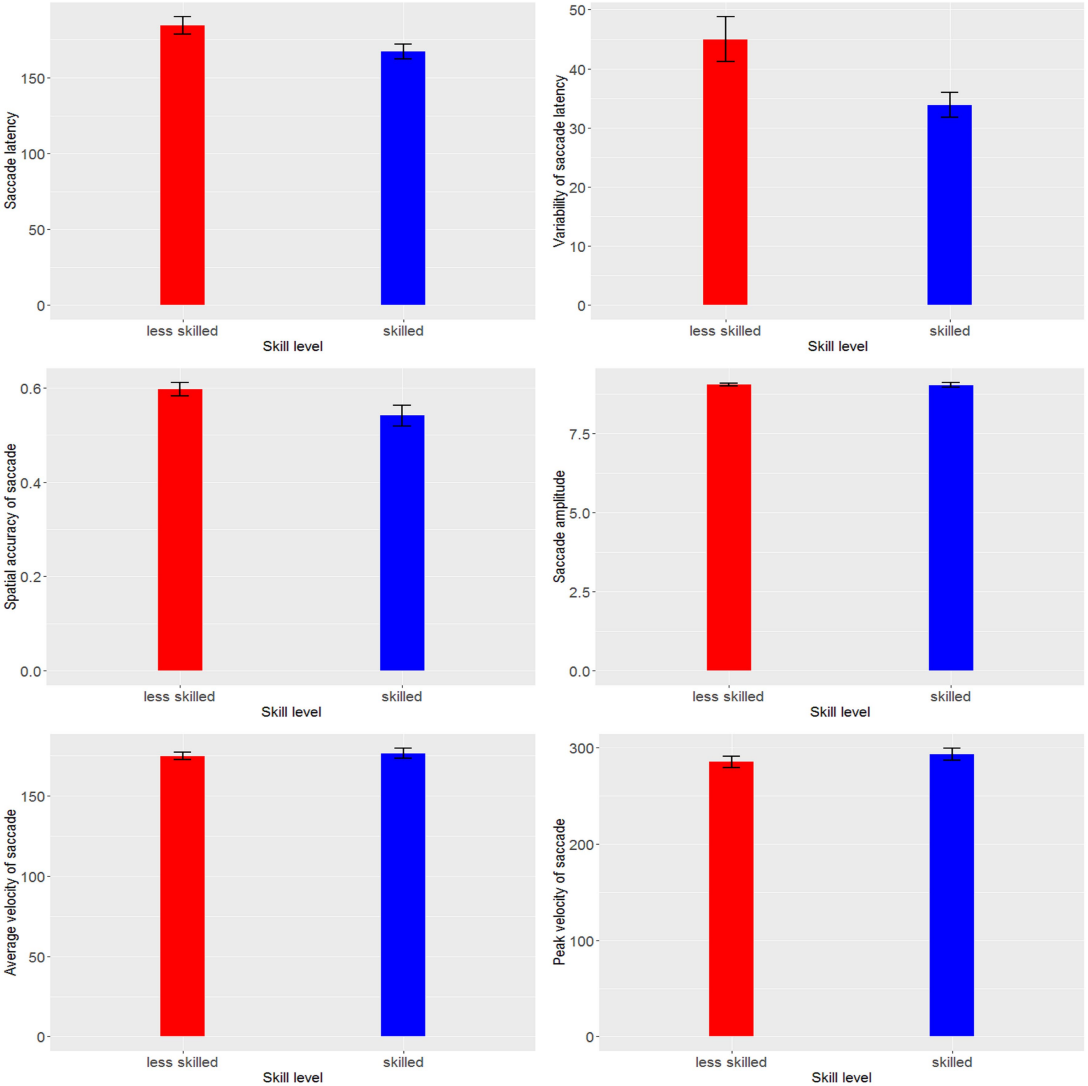
Differences in saccadic eye movement indexes between skill and less skilled players.

Independent sample *t* tests were conducted to compare the difference in each index between skilled and less skilled players (Table 2). Skilled players had shorter saccade latency (167.33ms, SD=23.32) than less skilled players (184.43ms, SD=32.02), *t*(54)=−2.21, *p*=0.031. Additionally, skilled players had decreased variability of saccade latency (33.89ms, SD=10.46) than less skilled players (45.00ms, SD=24.30), *t*(54)=−2.57, *p*=0.013. Skilled players showed higher spatial accuracy of saccade (0.54°, SD=0.11) than less skilled players (0.60°, SD=0.08), *t*(54)=−2.20, *p*=0.032. The saccade amplitude, peak velocity, and average velocity of two groups of players were comparable (|*t*|s<0.91; see Figure 1).

**Table 2:** Mean (SD) of saccadic eye movement indexes in prosaccade task for two groups of players.

Indexes	Skilled players	Less skilled players
Saccade latency (ms)	167.33 (23.32)	184.43 (32.02)
Variability of saccade latency (ms)	33.89 (10.46)	45.00 (24.30)
Saccade amplitude (°)	9.02 (0.35)	9.04 (0.26)
Spatial accuracy of saccade (°)	0.54 (0.11)	0.60 (0.08)
Peak velocity (°/s)	293.32 (29.56)	285.33 (34.21)
Average velocity (°/s)	176.43 (15.02)	174.80 (12.05)

## Discussion

In the current study, we conducted prosaccade task to investigate the difference in saccadic eye movements between skilled and less skilled female adolescent soccer players. As we hypothesized, the results showed that the skilled players, as compared to their less skilled counterparts, were faster to saccade to the target appeared peripherally, exhibited less variability of saccade latency, and more accurate saccade. Hence, our study supports the view that skilled players have higher cognitive ability and would thus perform better than less skilled players in prosaccade task.

The result that skilled players had shorter saccade latency than less skilled players suggests that skilled players had faster information processing speed than less skilled players. This is consistent with previous studies, demonstrating that saccade latency is influenced by information processing speed (Noiret et al., 2017, 2018) and others showing that saccade latency varies as a function of cognitive function (Coors et al., 2021; Thomas et al., 2021). More importantly, this result is also consistent with studies, demonstrating that players’ cognitive capacities vary as a function of their skill levels (Vestberg et al., 2012, 2017; Glavaš, 2020).

Another critical result is that less skilled players exhibited larger variability of saccade latency than skilled players. Kapoula et al. (2010) suggested that variability of saccade latency could be used as a good index of attentional fluctuation in saccadic eye movement tasks. This is due to more attentional lapses caused by more attentional fluctuation in one’s attentional system would contribute more longer reaction times in cognitive tasks (Schmiedek et al., 2007; McVay and Kane, 2012; Unsworth and Robison, 2016). This conclusion was supported by several studies, finding that individuals with inferior attentional system had larger variability of saccade latency than their counterparts with superior attention system (Mostofsky et al., 2001; Jazbec et al., 2006; Yang et al., 2013; Kiefer et al., 2018). Taken together, this result may imply that the attentional system of skilled soccer player is superior which leads to smaller attention fluctuation and less attentional lapse.

The result also suggests that skilled soccer players’ saccades are more accurate than less skilled soccer players. Previous studies have found that the spatial accuracy of prosaccade is mainly influenced by cerebellar (Ettinger et al., 2005; Optican, 2005; Coors et al., 2021), and the cerebellum, particularly the vermis, plays an important role in the spatial accuracy of saccade (Robinson and Fuchs, 2001; Leigh and Kennard, 2004). Hence, this result may imply that the function of cerebellum of skilled soccer player is superior to that of less skilled soccer. Moreover, this result also implies potential structural differences in brain underlying saccadic eye movement between skilled and less skilled soccer players.

No significant differences were observed in saccade amplitude, peak velocity, and average velocity. For saccade amplitude, previous study found that the saccade amplitude was mainly influenced by target eccentricity in prosaccade task (Petrella et al., 2019). On the one hand, the distance between the peripheral target and the position of the onset of saccade (fixation cross) is constant in the present experiment. On the other hand, all participants in this study had normal or corrected-to-normal vision. Therefore, the absent of significant difference between two groups in saccade amplitude is understandable. For saccade velocity, previous research has demonstrated that the peak velocity was relatively stable (Coors et al., 2021). Sparks (2002) suggested that, from the perspective of neurophysiology, peak velocity is subject to the number of spikes and firing rate of saccadic burst cells. Some researcher suggested that as people conduct approximately 200,000 saccades per day, the saccadic burst cells are continuously trained (Pratt and Welsh, 2006). Considering that all players in two groups had not suffered any neurological or psychiatric disorders or traumatic brain injury, their nervous system and oculomotor system should be normal and comparable. Therefore, it is also understandable that skilled and less skilled players did not differ in saccade velocity.

The findings of the present study shed some light on understanding of the relationship between saccadic eye movement and skill level in the field of sport and expertise. Besides, these findings supported the results of previous studies, indicating that cognitive ability differed as a function of skill level (Fontani et al., 2006; Brams et al., 2019; Barrett et al., 2020). Furthermore, the present study implies some practical applications for determining or predicting soccer players’ skill level and cognitive abilities by analyzing their saccadic eye movements during some eye movement tasks. It would be more economical and convenient for coaches and training instructors to determine a player’s skill level using some specific eye movement tasks.

Nevertheless, owing to the restriction of conditions, we should note that the present study has some limitations. For example, the current sample is representative of an adolescent, female, and soccer player sample. It remains unclear whether the results are fully generalizable to adolescent male or adult soccer players’ samples. Likewise, it is also unclear whether the results are fully generalizable to players in other sports. Besides, the relatively small sample was used in the present study as was subject to some objective circumstances. The future studies should include a larger sample to get greater statistical power to detect any differences between groups in cognitive processes on eye movements. Additionally, the current study only explored the differences in saccadic eye movements between skilled and less skilled soccer players using prosaccade task. Yet, growing body of research has shown that antisaccade task is a useful tool to examine the differences in inhibitory control and executive functions among different groups of population (Noiret et al., 2018; Gallagher et al., 2020; Bey et al., 2021; Coors et al., 2021). Therefore, future research should consider combining prosaccade task with antisaccade task when examining the saccadic eye movements of soccer players.

## Conclusion

In summary, the results of the current study suggest that skilled soccer players outperform less skilled soccer players on saccadic eye movement in prosaccade task indicating superior cognitive processes underlying saccadic eye movement in skilled soccer players. Overall, the present findings have implications for understanding the relationship between saccadic eye movements and skill level.

## Data Availability Statement

The original contributions presented in the study are included in the article/Supplementary Material, further inquiries can be directed to the corresponding author.

## Ethics Statement

The studies involving human participants were reviewed and approved by Academic and Ethics Committee of the Fujian Normal University. Written informed consent to participate in this study was provided by the participants’ legal guardian/next of kin.

## Author Contributions

ZJ contributed to the conception, design of the study, conducted the experiment, performed the statistical analysis, and wrote the first draft of the manuscript.

## Funding

This research was supported by a grant from the Fujian Social Science Foundation (FJ2018C064), and by a grant from the Planning Project of Fujian Provincial Department of Education (JAS180075).

## Conflict of Interest

The author declares that the research was conducted in the absence of any commercial or financial relationships that could be construed as a potential conflict of interest.

## Publisher’s Note

All claims expressed in this article are solely those of the authors and do not necessarily represent those of their affiliated organizations, or those of the publisher, the editors and the reviewers. Any product that may be evaluated in this article, or claim that may be made by its manufacturer, is not guaranteed or endorsed by the publisher.
